# Multiple Convergent Origins of Workerlessness and Inbreeding in the Socially Parasitic Ant Genus *Myrmoxenus*

**DOI:** 10.1371/journal.pone.0131023

**Published:** 2015-07-29

**Authors:** Jürgen Heinze, Alfred Buschinger, Theo Poettinger, Masaki Suefuji

**Affiliations:** 1 Zoology / Evolutionary Biology, University of Regensburg, Regensburg, Germany; 2 Professor Emeritus of TU Darmstadt, Rossbergring 18, Reinheim, Germany; Universidade de São Paulo, BRAZIL

## Abstract

The socially parasitic ant genus *Myrmoxenus* varies strongly in fundamental life history traits, such as queen-worker ratio, the timing of sexual production, and mating behavior. *Myrmoxenus* queens generally take over nests of *Temnothorax* ants, kill the resident queen by throttling, and force the workers to take care of the social parasite’s brood. Young queens of *M*. *ravouxi* and other species produce large numbers of workers, which during “slave-raids” pillage host pupae from neighboring *Temnothorax* colonies to increase the workforce in their own nests. Other species, such as *M*. *corsicus*, have lost caste polyphenism and rear only male and female sexual offspring. Using sequences of the genes CO I / CO II and wingless we reconstruct the phylogeny of *Myrmoxenus* and document that the worker caste was lost convergently at least three times. Furthermore, mating in the nest and inbreeding obviously also evolved in parallel from ancestors whose sexuals presumably mated during nuptial flights. *Myrmoxenus* might thus provide a suitable model to investigate caste differentiation and the plasticity of mating behavior in Hymenoptera.

## Introduction

The enormous ecological success of ants, bees, and wasps is based on their efficient division of labor among the two female castes [1, 2]. Reproductive queens and non-reproductive workers are well adapted to their respective tasks and they often differ strikingly in size and morphology, with ant queens often being much larger and more long-lived than their workers. Though recent studies have revealed a more or less pronounced influence of genotype on caste differentiation, whether a female larva grows into a worker or a queen is typically controlled by the environment [3], making caste diphenism one of the most conspicuous and abundant cases of phenotypic plasticity.

In particular in ants, the ancestral pattern of queen-worker diphenism has evolved additional, more or less complex modifications. For example, in leaf-cutting ants, *Atta*, worker-destined larvae may develop into different worker morphs, which behaviorally specialize in leaf-cutting, leaf transportation, defense, or fungus processing etc. [4]. Similarly, queens with reduced wings and simplified thoracic structures may complement the “normal” queens, which at least early in their adult lives are endowed with wings and capable of active flight [5–7].

Other ants show exactly the opposite trend, i.e., a reduction of the number of reaction norms of female larvae. On the on hand, morphological queens have been completely replaced by reproductive workers in a number of Ponerinae [8]. On the other hand, several species of socially parasitic ants have lost the worker caste, i.e., parasite queens rely on host workers from another species to rear the male sexuals and young queens of the parasite species [9–11]. Monophyla, in which queens produce both workers and sexuals in some species and only sexuals in others, might be ideal model systems to elucidate the genomic basis of queen-worker diphenism and to better understand the evolutionary dynamics of caste ratios. *Myrmoxenus*, a genus famous for the bizarre founding behavior of its queens, provides a particularly promising example of such variation. *Myrmoxenus* queens sneak into colonies of *Temnothorax* ants, where they eliminate the resident queen by slowly throttling it with their mandibles for days or weeks. Of the 11 presently recognized species of this genus, at least six are active slave-makers [12]: the queens of these species produce workers, which instead of engaging in the daily maintenance activities of the colony pillage pupae from neighboring *Temnothorax* nests to replenish or increase the own stock of host workers [13]. *M*. *kraussei* is a “degenerate slave-maker,” i.e., worker number is greatly reduced and though slave raids have been elicited in the lab it was thought to be unlikely that they occur in nature [14] (but see [15]). Finally, at least three taxa have completely lost the worker caste and queens produce only female and male sexuals. Variation in caste ratio is associated with additional variation in mating behavior (mating flights vs. intranidal mating, i.e., mating in the nest) and the pattern of brood production [12]. Based on the detailed description and analysis of the various life histories evolutionary pathways linking the various taxa were proposed [12].

Aim of our present study is to determine how the different life histories of *Myrmoxenus* are interrelated, i.e., whether the worker caste and mating flights were lost once or multiply in evolution. We therefore constructed a phylogeny of this genus based on sequences of mitochondrial CO I / CO II and the nuclear gene wingless and use it to reconstruct the evolution of the various life history traits of this fascinating genus.

## Methods

### Taxon sampling

We here chose to keep *Myrmoxenus* as a separate genus rather than synonymize it with *Temnothorax*, as recently suggested [16]. In our opinion, the presently available data are not sufficient to clearly document if and where *Myrmoxenus* is rooted within the large and heterogeneous genus *Temnothorax*. The phylogeny in [16] contains only two representatives of the probably 600 extant species of *Temnothorax* [17]: *T*. *rugatulus* from North America and *T*. *poeyi* from Cuba. In particular the latter species differs strongly from Palearctic *Temnothorax*. It was originally described as belonging to a separate genus, *Macromischa* [18] and later referred to as *Croesomyrmex poeyi* [19]. A preliminary phylogeny of *Myrmoxenus* and several old-world *Temnothorax* based on 651 bp of CO I did not resolve the basal branching pattern and thus neither supports nor rejects paraphyly of *Temnothorax* (J. Beibl and J. Heinze, unpubl.). According to [17] a taxonomy that considers morphological, biochemical, and ethological characters is preferable to a categorization exclusively based on genetic sequences and the chronology of speciation events. This view is certainly controversial but progressively finds more and more adherents [20]. We leave it to others to work on the Pandora’s box of evolutionary versus cladistic classifications, but in the light of our limited knowledge of the relationships among the taxa of the tribe Formicoxenini we suggest to keep the socially parasitic lineages as separate genera. *Myrmoxenus* is an easily recognizable, rapidly evolving monophylum with distinctive characters, such as a strongly developed ventral petiolar lobe, and the unique behavior of throttling host queens, and keeping it as a separate genus separate from *Temnothorax* thus is more informative about evolutionary processes.

The current study expands an earlier research with *M*. *tamarae* [21], *M*. *kraussei*, and *M*. *ravouxi* [22] by now including 10 of the 12 currently recognized species of *Myrmoxenus* (S1 Table). No material was available of *M*. *africanus* from Algeria and *M*. *zaleskyi* from Slovakia. Most samples had been collected by A. Buschinger in the 80s and 90s and were thereafter stored in 75% EtOH / 2% glycerol. Nuclear and also mitochondrial DNA was frequently degraded and clear sequences were not available from all samples. Furthermore, preliminary investigations had shown that nuclear genes vary little among the different species (maximum difference between *T*. *recedens* and *M*. *ravouxi*: abdominal-A: 5 of 404 bp, 1.2%; longwave rhodopsin: 11 of 541 bp, 2.0%; elongation factor 1α-F1: 7 of 376bp, 1.9%; see also [21]). We therefore concentrated on wingless, which could be amplified most consistently, was more variable, and has been shown previously to be informative for species- and genus-level analyses (e.g., [23, 24]). Unfortunately, wingless sequences could not be obtained from all samples. The mtDNA sequence of *M*. *algerianus* from Mts. de Belezma contained several ambiguous nucleotides and we therefore added a shorter sequence from GenBank to the analysis. Two common host species of *Myrmoxenus*, *T*. *recedens* and *T*. *unifasciatus*, were included as outgroups. Species names are based on morphology.

### DNA sequencing and phylogenetic analysis

Genomic DNA was extracted using a cetyltrimethyl ammonium bromide (CTAB) protocol [25]. In this study we amplified the nuclear gene wingless (WG, approximately 350 base pairs) and the mitochondrial gene cytochrome oxidase subunit I/II (COI/II, approximately 1450 bp, including tLeu-region) using the primer pairs C1-J-2195 [26] and CW-3031rev [27] and COI750out and C2-N-3661 [26]. For both genes, PCR was carried out with Mastercycler (Eppendorf AG, Hamburg) using a total reaction volume of 20μl. The mix consisted of 1μl template DNA, 1μl 1U/μl Taq polymerase (Fermentas), 1.2μl 10μM of each forward and reverse primer (MWG Biotech), 2μl 10x Taq buffer (containing 100mM Tris-HCl pH 8.8, 500mM KCl, 0.8% Nonidet P40; MBI Fermentas), 4μl 5x Enhancer (PEQLAB), 1.6μl 25mM MgCl2, 0.25μl 100mM of mix of each dNTP (Fermentas, St. Leon-Rot) and 7.75μl PCR water. PCR cycling program for wingless consisted of 40cycles of 30sec at 95°C (denaturation), 30sec at 55°C (annealing) and 30sec at 72°C (extension), preceded by 3min at 95°C and followed by a final extension of 7min at 72°C and for mitochondrial gene COI/COII of 40cycles of 1min at 94°C, 1min at 50°C and 1min30sec at 72°C, preceded by 3min at 94°C and followed by a final extension of 6min at 72°C.

The successful PCR products were purified with the High Pure PCR Cleanup Micro Kit (Roche Diagnostics GmbH, Mannheim). Cycle sequencing was then conducted in a total reaction volume of 20μl with 2μl of Big Dye Terminator v1.1 Sequencing RR-100 (Applied Biosystems, Weiterstadt), 3μl of 5x sequencing buffer, 1μl 10μM of each primer, 2–7μl of the purified product and 7–12μl of PCR-H2O. Cycling program consisted of 30 cycles of 10sec at 96°C, 8sec at 55°C (for wingless)/ 50°C (for mitochondrial gene COI/COII) and 4min at 60°C. After cleaning all products were sequenced with ABI Prism 310 Genetic Analyzer (Applied Biosystems, Weiterstadt), and all sequences were corrected with Sequencing Analysis 3.4 and aligned with Bio-Edit 7.0.5.2 [28] using the Clustal W algorithm. All sequences were verified by analysis of two nestmates for each sample.

Phylogeny was restricted using a Bayesian analysis with MrBayes version 3.2.2 [29] and maximum likelihood analysis with RAxML v.7.4.2 [30]. For drawing the figures we used FigTree version 1.3.1 (available at http://tree.bio.ed.ac.uk/software). The model of evolution for all genes was estimated with JModeltest v.2.1.3 [31]. We assigned the evolutionary model using the Akaike information criterion for each partition, GTR+G+I for CO I / CO II and HKY for WG. We conducted the MrBayes analysis with the default settings of four Markov chains (three heated, one cold) and the heating parameter set at 0.2. Each analysis was conducted with a Markov Chain Monte Carlo method with 3Mio generations, sampled every 1000 generations. The consensus tree was created discarding the first 25% of the sampled trees (the burn-in). The maximum likelihood analysis was conducted under a GTR+G+I model for the concatenated data set and fast bootstrapping with 1000 replicates.

No permits were required for our study. All experiments comply with national and international law.

## Results

Within *Myrmoxenus*, GC content of CO I / CO II ranged from 28.8% (*M*. *bernardi*) to 30.3% (*M*. *birgitae*), with a median of 29.6%, that of wingless from 63.1 (*M*. *tamarae)* to 63.9% *(M*. *gordiagini*), with a median of 63.7%. The alignment included 328 variable sites and 239 parsimony informative sites in CO I / CO II and 16 variable sites in wingless, of which 8 were parsimony informative. The largest differences were found between *T*. *recedens / T*. *unifasciatus* and *M*. *ravouxi* (247 and 251 of 1430 bp of CO I / CO II, 17.3% and 17.5%, 8 differences in 355 bp of wingless, 2.3%). GenBank accession numbers are given in S1 Table.

**Fig 1:**
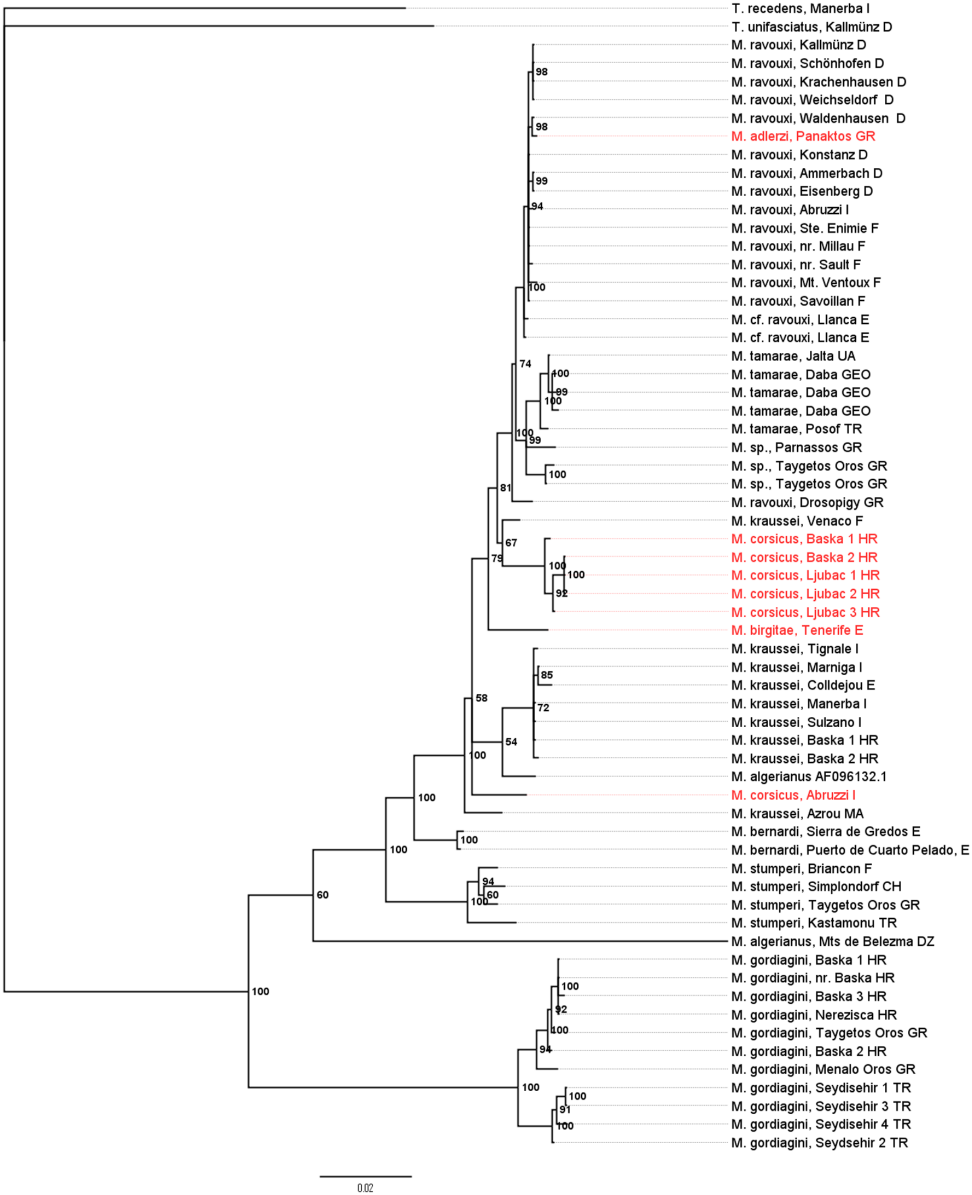
Bayesian tree of the socially parasitic ant genus *Myrmoxenus* inferred from sequences of the nuclear gene wingless and the mitochondrial gene CO I / CO II. Bayesian posterior probabilities (as percentages) are given at the nodes. Workerless species are given in red.

**Fig 2:**
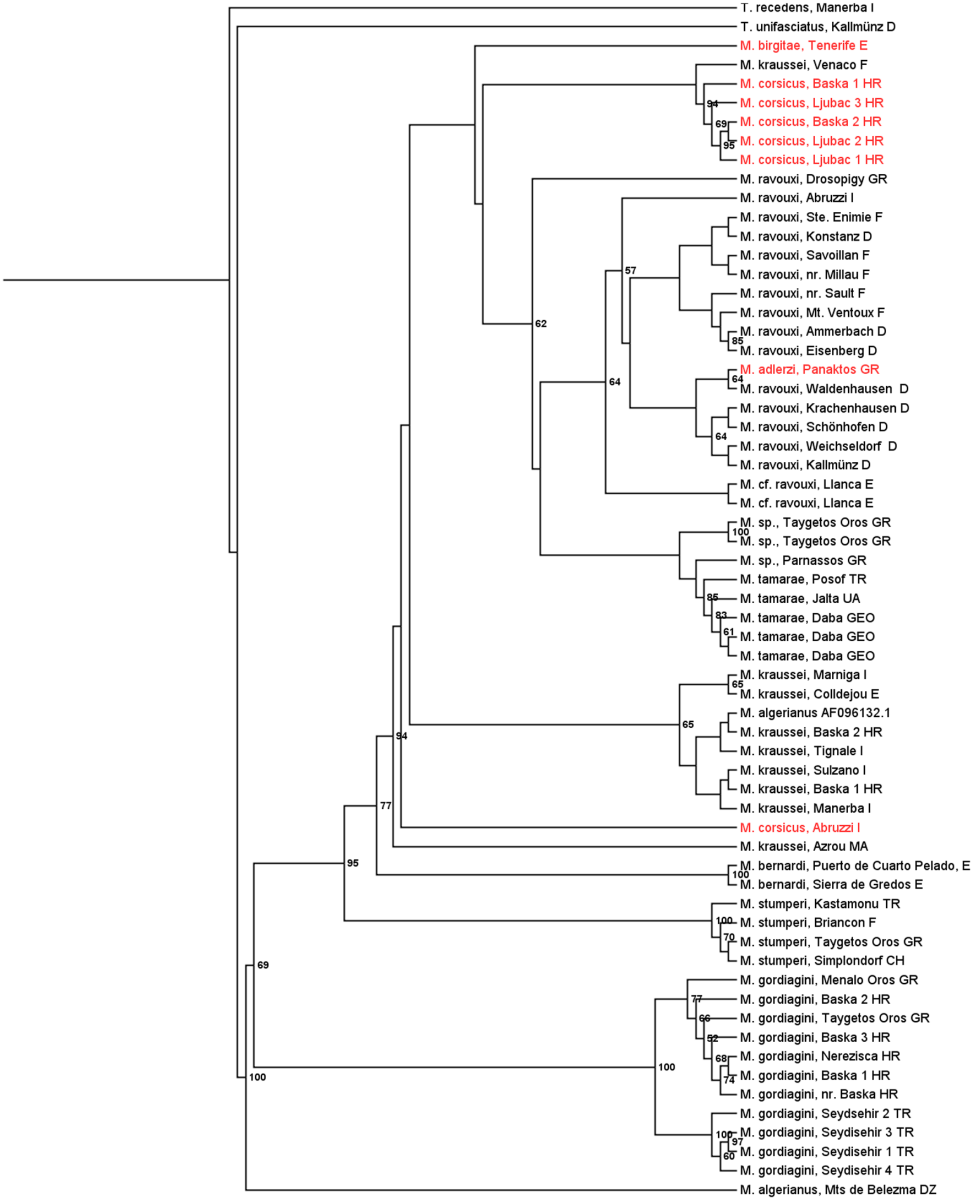
Maximum likelihood tree of the socially parasitic ant genus *Myrmoxenus* inferred from sequences of the nuclear gene wingless and the mitochondrial gene CO I / CO II. Figures at the nodes represent the percentage of replicate trees with a particular branching pattern (only > 50%). Workerless species are given in red.

Both Bayesian (Fig 1) and maximum likelihood analyses (Fig 2) of the concatenated sequences of wingless and COI/COII resulted in similar, robust tree topologies with Bayesian posterior probabilities of 1 for several of the more basal major nodes. *M*. *gordiagini* consistently formed a well-supported clade that split early from the other *Myrmoxenus* lineages. Of the remaining taxa, *M*. *stumperi* and *M*. *bernardi* are well-separated from the other lineages. The other taxa form two clusters, A) with *M*. *kraussei* and *M*. *algerianus*, and B) with *M*. *ravouxi*, *M*. *tamarae* and the three workerless species *M*. *adlerzi*, *M*. *birgitae*, and *M*. *corsicus*, with a few inconsistent placements. For example, specimens from Algeria and Italy, by morphology assigned to *M*. *kraussei* and *M*. *corsicus*, form the outgroup to the two branches A and B, and one sample referred to as *M*. *kraussei* from Venaco clusters with clade A. Because of the morphological similarities among the different taxa we cannot exclude that this presents incorrect determination and that some of the “misplaced” specimens in fact present undescribed species. The placement of material assigned to *M*. *algerianus* from Mts de Belezma, Algeria, as outgroup to other *Epimyrma* is presumably erroneous as the sequence contained a number of ambiguous positions.

A phylogeny based only on the nuclear gene wingless did not help to solve these apparent “misplacements”. While it supported *M*. *gordiagini*, *M*. *tamarae*, *M*. *bernardi*, and *M*. *stumperi* as separate lineages with Bayesian probabilities of > 0.9, it did not distinguish between *M*. *ravouxi* and *M*. *kraussei*. We could not amplify wingless in the workerless species.

## Discussion

Our phylogeny of the socially parasitic ant genus *Myrmoxenus* reveals the existence of two well-supported lineages, one with *M*. *gordiagini* and the other with the remaining taxa. This matches the original taxonomic treatment of all species other than *M*. *gordiagini* as members of a separate genus, *Epimyrma*. The two genera were merged first as *Epimyrma* [32] and later, because of priority, as *Myrmoxenus* [33]. Within the *“Epimyrma”-*clade, one or two species *(M*. *stumperi*, *M*. *bernardi*) are rather well supported. The remaining lineage apparently has undergone a relatively recent range expansion and radiation, which makes it difficult to clearly resolve its phylogeny (*M*. *adlerzi*, *M*. *algerianus*, *M*. *birgitae*, *M*. *corsicus*, *M*. *kraussei*, *M*. *ravouxi*, *M*. *tamarae*). Recent divergence is also supported by the observation that several taxa of “*Epimyrma*,*”* though strictly isolated in nature, readily hybridize and produce fertile offspring in the lab [34, 35]. In contrast, cross-breeding of *M*. *kraussei* and *M*. *gordiagini* did not yield any progeny [35] (no mating experiments were done with *M*. *stumperi*). The specialization of most taxa on particular host species, together with the frequent replacement of mating flights by sib-mating in the nest might promote speciation and prevents natural hybridization (see also [36]]. The observed “incorrect” placements of some taxa (e.g., *M*. *“corsicus”* from Abruzzi or *M*. *“kraussei”* from Azrou) might reflect incomplete lineage sorting in this rapidly diverging genus or the existence of previously not recognized novel species.

Despite of the shallow branching pattern and the predominance of mtDNA data in the analyses, the trees allow the tentative reconstruction of the evolution of the different life histories and colony demographies of *Myrmoxenus*. The workerless taxa obviously do not form a monophyletic cluster, but workers were lost convergently in *M*. *adlerzi* within *M*. *ravouxi*, in *M*. *birgitae* and *M*. *corsicus* as sister taxon of *M*. *ravouxi*, and in *M*. “*corsicus*” from Abruzzi as a third separate lineage. A further independent origin of workerlessness is suggested by the observation that worker number varies tremendously within *M*. *kraussei*, with one population from Crete having lost workers [37] (no molecular data available). The stable production of queens from all female brood regardless of social and environmental conditions suggests an extensive loss of phenotypic plasticity. The sporadic occurrence of workers in naturally workerless species in the lab (e.g., one worker in a total of 745 female offspring of *M*. *adlerzi*, [38]) indicates the retention of cryptic plasticity rather than genetic accommodation. Though numerous socially parasitic ants have lost the worker caste, the close relatedness among *Myrmoxenus* species with and without workers and the large variation of queen/worker ratios even within single species make this genus a suitable model to investigate the functional genomics of caste differentiation in ants.

One might assume that workerless parasites with mating in the nest evolved from slave-maker species, which already had switched from nuptial flights to mating in the nest, such as *M*. *kraussei* or *M*. *bernardi* [35]. Our phylogeny does not support this obvious view. Instead, workerless *M*. *adlerzi*, *M*. *birgitae*, and *M*. *corsicus*, all with intranidal mating, appear to have evolved within the clade of *M*. *ravouxi*, a widespread species in which mating is thought to occur during nuptial flights. An excess of homozygous genotypes in several populations of *M*. *ravouxi* nevertheless suggests a mixed mating system with occasional sib-mating [22]. Mating behavior therefore appears to be a rather labile trait and nuptial flights that guarantee outbreeding have been convergently replaced by exclusive intranidal mating among close relatives in both species with and without workers. The driving force of this change of the mating location is the difficulty of finding mates when population sizes are low (e.g., [12, 39]). Queens, which mated in their natal nests, disperse on foot and usurp host colonies nearby. Because of local resource competition this selects against slave-raids and promotes the loss of workers [12].

Inbreeding is particularly detrimental for species with haplodiploidy and single-locus complementary sex determination, the typical mode of sex determination in the social Hymenoptera, because it results in the production of inviable or sterile diploid males. Breeding experiments in *Myrmoxenus* with intranidal mating did not yield diploid males, suggesting that the convergent transition to mating in the nest is associated with a change in the mechanism of sex determination [12]. This again makes *Myrmoxenus* an interesting system to study the evolution of sex determination in social Hymenoptera, which appears to be more variable than occasionally assumed (see also [40, 41].

Finally, our phylogeny indicates that the production of sexuals from non-overwintering brood (“rapid brood”) evolved convergently in *M*. *kraussei*, *M*. *corsicus*, and *M*. *adlerzi*, while sexual larvae of the other taxa hibernate in the natal nest before pupation [12]. Rapid brood may be an adaptation to worker loss, because the absence of slave-raids limits the life expectancy of the parasite colony to that of the host workers present during usurpation. Furthermore, it allows a very quick exploitation of a local patch of host nests.

At present it remains unclear which ecological factors triggered the evolution of the loss of workers, but climate might be important. Most workerless taxa have been collected in very dry and hot places with sparse vegetation cover (e.g., [37, 42], while the active slave-makers tend to occur either in more temperate woodland, alpine pastures, or, in the Mediterranean, in shady places in deciduous forests (e.g., [12, 43]. Slave raids are probably difficult in summer when they are most profitable because of the large numbers of worker pupae present in *Temnothorax* colonies. Host nests might be more difficult to locate because many host species protect themselves from desiccation and heat by moving deeper down in rock crevices or other nest materials. The observation that neighboring populations of *M*. *kraussei* greatly differ in average worker number indicates, however, that additional factors, e.g., microclimate or evolutionary history, might also play a role [44].

## Supporting Information

S1 Table Origin of the analyzed specimens of *Myrmoxenus* and GenBank accession numbers of the gene sequences. AB, JH, MS: authors of the present study; AS: Andreas Schulz, Leichlingen. Sequences with accession numbers LK392459 to LK392513 are from Gratiasvhili et al. (2014). The 400bp sequence of *M*. *algerianus* (AF096132) was submitted to GenBank by P. Douwes, B. Stille and M. Stille in 1999.
(PDF)

## References

[1] Hölldobler B, Wilson EO (1990) The Ants. Cambridge, Mass: Harvard University Press.

[2] Hölldobler B, Wilson EO (2008) The Superorganism: The Beauty, Elegance, and Strangeness of Insect Societies. New York: Norton & Co.

[3] Schwander T, Lo N, Beekman M, Oldroyd BP, Keller L (2010) Nature versus nurture in social insect caste differentiation. Trends Ecol Evol 25: 275–282. 10.1016/j.tree.2009.12.001 20106547

[4] Oster GF, Wilson EO (1978) Caste and Ecology in the Social Insects. Princeton, NJ: Princeton University Press. 740003

[5] Heinze J, Keller L (2000) Alternative reproductive tactics: a queen perspective in ants. Trends Ecol Evol 15: 508–512. 11114438 10.1016/s0169-5347(00)01995-9

[6] Peeters C (2012) Convergent evolution of wingless reproductives across all subfamilies of ants, and sporadic loss of winged queens (Hymenoptera: Formicidae). Myrmecol News 16: 75–91.

[7] Peeters C, Ito F (2001) Colony dispersal and the evolution of queen morphology in social Hymenoptera. Annu Rev Entomol 46: 601–630. 11112181 10.1146/annurev.ento.46.1.601

[8] Peeters C (1991) The occurrence of sexual reproduction among ant workers. Biol J Linn Soc 44: 141–152.

[9] Buschinger A (1990) Sympatric speciation and radiative evolution of socially parasitic ants—heretic hypotheses and their factual background. Z zool Syst Evolut forsch 28: 241–260.

[10] Buschinger A (2009) Social parasitism among ants: a review (Hymenoptera: Formicidae). Myrmecol News 12: 219–235.

[11] Bourke AFG, Franks NR (1991) Alternative adaptations, sympatric speciation and the evolution of parasitic, inquiline ants. Biol J Linn Soc 43: 157–178.

[12] Buschinger A (1989) Evolution, speciation, and inbreeding in the parasitic ant genus Epimyrma (Hymenoptera, Formicidae). J evol Biol 2: 265–283.

[13] Buschinger A, Ehrhardt W, Winter U (1980) The organization of slave raids in dulotic ants: a comparative study (Hymenoptera; Formicidae). Z Tierpsychol 53: 245–264.

[14] Buschinger A, Winter U (1983) Population studies of the dulotic ant, *Epimyrma ravouxi*, and the degenerate slavemaker, *Epimyrma kraussei* (Hymenoptera: Formicidae). Entomol Gener 8: 251–266.

[15] Suefuji M, Heinze J (2015) Degenerate slave-makers—but nevertheless slave-makers? Host worker relatedness in the ant *Myrmoxenus kraussei* . Integr Zool 10: 182–185. 10.1111/1749-4877.12120 25316159

[16] Ward PS, Brady SG, Fisher BL, Schultz TR (2014) The evolution of myrmicine ants: phylogeny and biogeography of a hyperdiverse ant clade (Hymenoptera: Formicidae). Syst Entomol 40: 61–81.

[17] Seifert B (2007) Die Ameisen Mittel- und Nordeuropas. Görlitz: Lutra

[18] Wheeler WM (1913) The ants of Cuba. Bull Mus Comp Zool Harvard Coll 54: 477–505.

[19] Wheeler WM (1931) New and little known ants of the genera *Macromischa*, *Croesomyrmex*, and *Antillaemyrmex* . Bull Mus Comp Zool Harvard Coll 72: 1–34.

[20] Hörandl E, Stuessy TF (2010) Paraphyletic groups as natural units of biological classification. Taxon 59: 1641–1653.

[21] Gratiashvili N, Bernadou A, Suefuji M, Seifert B, Heinze J (2014) The Caucaso-Anatolian slave making ant *Myrmoxenus tamarae* (Arnoldi, 1968) and its more widely distributed congener *Myrmoxenus ravouxi* (André, 1896): a multidisciplinary comparison (Hymenoptera: Formicidae). Org Div Evol 14: 259–267.

[22] Suefuji M, Heinze J (2014) The genetic population structure of two socially parasitic ants: the active slave-maker *Myrmoxenus ravouxi* and the "degenerate slave-maker" *M*. *kraussei* . Conserv Genet 15, 201–211.

[23] Schultz TR, Brady SG (2008) Major evolutionary transitions in ant agriculture. Proc Natl Acad Sci USA 105: 5435–5440. 10.1073/pnas.0711024105 18362345 PMC2291119

[24] Cardoso DC, Cristiano MP, Heinze J, Tavares MG (2014) A nuclear DNA based phylogeny of endemic sand dune ants of the genus *Mycetophylax* (Emery, 1913): how morphology is reflected in molecular data. Mol Phylogenet Evol 70: 378–382. 10.1016/j.ympev.2013.10.012 24161832

[25] Sambrook J, Russell D (2001) Molecular Cloning, 3rd edn New York: Cold Spring Harbor Laboratory Press

[26] Simon C, Frati F, Benckenbach A, Crespi B, Liu H, Flook P (1994) Evolution, weighting, and phylogenetic utility of mitochondrial gene sequences and a compilation of conserved polymerase chain reaction primers. Ann Entomol Soc Am 87: 652–701.

[27] Beibl J, Stuart RJ, Heinze J, Foitzik S (2005) Six origins of slavery in formicoxenine ants. Insectes Soc 52: 291–297.

[28] Hall TA (1999) BioEdit: a user-friendly biological sequence alignment editor and analysis program for Windows 95/96/NT. Nucl Acids Symp Ser 41: 95–98.

[29] Ronquist F, Teslenko M, van der Mark P, Ayres DL, Darling A, Höhna S, et al (2012) MrBayes 3.2: efficient Bayesian phylogenetic inference and model choice across a large model space. Syst Biol 63: 539–542. 10.1093/sysbio/sys029 PMC3329765 22357727

[30] Stamatakis A (2006) RAxML-VI-HPC: maximum likelihood- based phylogenetic analyses with thousands of taxa and mixed models. Bioinformatics 22: 2688–2690. 16928733 10.1093/bioinformatics/btl446

[31] Darriba D, Taboada GL, Doallo R, Posada D (2012) jModelTest 2: more models, new heuristics and parallel computing. Nat Methods 9: 772. 10.1038/nmeth.2109 PMC4594756 22847109

[32] Bolton B (1994) Identification guide to the ant genera of the world. Cambridge, Mass: Harvard University Press.

[33] Schulz A, Sanetra M (2002) Notes on the socially parasitic ants of Turkey and the synonymy of *Epimyrma* (Hymenoptera: Formicidae). Entomofauna 23: 157–172.

[34] Jessen K, Klinkicht M (1990) Hybridization in the social parasitic ant genus *Epimyrma* (Hymenoptera, Formicidae). Insectes Soc 37: 273–293.

[35] Buschinger A (2001) Multiple Hybridisierung von Arten der Ameisen-Gattung *Epimyrma* (Hymenoptera: Formicidae), und Beobachtung zur Ausbeutung der Wirtsarten durch die Parasiten. Myrmecol Nachr 4: 25–35.

[36] Beibl J, d'Ettorre P, Heinze J (2007) Cuticular profiles and mating preference in a slave-making ant. Insectes Soc 54: 174–182.

[37] Buschinger A (1989). Workerless *Epimyrma kraussei* Emery 1915, the first parasitic ant of Crete. Psyche 96: 69–74.

[38] Douwes P, Jessen K, Buschinger A (1988) *Epimyrma adlerzi* n. sp. (Hymenoptera: Formicidae) from Greece: morphology and life history. Entomol Scand 19: 239–249.

[39] Wilson EO (1963) Social modifications related to rareness in ants. Evolution 17: 249–253.

[40] Van Wilgenburg E, Driessen G, Beukeboom LW (2006) Single locus complementary sex determination in Hymenoptera: an "unintelligent" design? Front Zool 3: 1 16393347 10.1186/1742-9994-3-1 PMC1360072

[41] Schrempf A, Aron S, Heinze J (2006) Sex determination and inbreeding depression in an ant with regular sib-mating. Heredity 97: 75–80. 16705320 10.1038/sj.hdy.6800846

[42] Buschinger A, Winter U (1985) Life history and male morphology of the workerless parasitic ant *Epimyrma corsica* (Hymenoptera: Formicidae). Entomol Gener 10: 65–75.

[43] Buschinger A, Winter U, Faber W (1983) The biology of *Myrmoxenus gordiagini* Ruzsky, a slave-making ant (Hymenoptera, Formicidae). Psyche 90: 335–342.

[44] Buschinger A, Fischer K, Guthy H-P, Jessen K, Winter U (1986) Biosystematic revision of *Epimyrma kraussei*, *E*. *vandeli* and *E*. *foreli* (Hymenoptera: Formicidae). Psyche 93: 253–276.

