# Supplementary material for: Multiple Convergent Origins of Workerlessness and Inbreeding in the Socially Parasitic Ant Genus Myrmoxenus
Source: PLoS One. 2015 Jul 29;10(7):e0131023. doi: 10.1371/journal.pone.0131023 (PMC4519230; doi:10.1371/journal.pone.0131023)
Supplement: S1 Table — AB, JH, MS: authors of the present study; AS: Andreas Schulz, Leichlingen. Sequences with accession numbers LK392459 to LK392513 are from Gratiasvhili et al. (2014). The 400bp sequence of M. algerianus (AF096132) was submitted to GenBank by P. Douwes, B. Stille and M. Stille in 1999. (PDF) [file pone.0131023.s001.pdf]

Species[1]	Collecting site	Collection number and year	CO I / CO II Accession number	Wingless Accession number
Temnothorax unifasciatus	Kallmünz, D	J. Heinze (2008)	LK392516	LK392481
M. adlerzi	Panaktos, GR	AB 12326 (1985)	LK392506	
M. birgitae	Tenerife, Valle de Orotava, E	AB 14426 (1990)	LN866949	
M. corsicus	Baška, HR	AB 12405 (1985)	LN866950	
M. corsicus	Ljubac, HR	AB 11.659 (1983)	LN866951	
M. corsicus	Abruzzi, Civitella, I	AB 14549 (1990)	LN866952	
M. kraussei	Venaco, Corsica, F	AB 10382 (1982)	LN866953	
M. kraussei	Tignale, Lago di Garda, I	D. Ortius IT-6 (2001)	LN866954	LN866968
M. kraussei	Marniga, Lago di Garda, I	J. Beibl I-153 (2003)	LN866955	LN866969
M. kraussei	Manerba, Lago di Garda, I	J. Beibl I-137 (2003)	LN866956	LN866971
M. kraussei	Sulzano, Lago d'Iseo, I	J. Beibl, I-170 (2003)	LN866957	LN866972
M. kraussei	Baška 1, HR	J. Beibl, K-36 (2005)	LN866958	LN866973
M. kraussei	Azrou, MA	AB 13127 (1987)	LN866959	
M. ravouxi	Abruzzi, Civitella, I	AB 14525 (1990)	LK392496	LK392467
M. ravouxi	Ammerbach (Jena), D	J. Trettin	LK392493	LK392464
M. ravouxi	Drosopigi, GR	AB 12359 (1985)	LK392497	
M. ravouxi	Eisenberg, A	AB 15592 (1997)	LK392495	LK392466.
M. ravouxi	Kallmünz, D	MS (2011)	LK392488	LK392468
M. ravouxi	Konstanz, D	H. Martz (2009)	LK392490	LK392462
M. ravouxi	Krachenhausen, D	MS (2011)	LK392492	LK392465
M. ravouxi	Mt. Ventoux, F	AB 13894 (1988)	LK392498	LK392472
M. ravouxi	nr. Millau, Gorges du Tarn, F	AB 16163 (2003)	LK392499	LK392470
M. ravouxi	nr. Sault, F	AB 13908 (1988)	LK392500	LK392471
M. ravouxi	Savoillan, F	J. Beibl, F-48 (2003)	LK392501	LK392473
M. ravouxi	Schönhofen, D	MS (2009)	LK392491	LK392463
M. ravouxi	St. Enimie, Gorges du Tarn, F	AB 16168 (2003)	LK392502	LK392469
M. ravouxi	Waldenhausen, D	MS (2008)	LK392489	LK392461
M. ravouxi	Weichseldorf, D	MS (2011)	LK392494	LK392474
M. sp.	Llanca, E	AB 16154 (2003)	LK392510	LK392511
M. sp.	Parnassos, GR	AB 15477 (1996)	LK392512	
M. sp.	Taygetos Oros, GR	AS 0319 (2011)	LK392514	LK392459

M. sp.	Taygetos Oros, GR	AS 0332 (2011)	LK392515	LK392460
M. tamarae	Jalta, UA	AB 15432 (1995)	LK392509	LK392477
M. tamarae	Posof, TR	AS 0310 (2012)	LK392513	
M. tamarae	Daba I, GEO	JH GEO I-3 (2010)	LK392503	LK392478
M. tamarae	Daba 2, GEO	JH GEO I-4 (2010)	LK392504	LK392479
M. tamarae	Daba 3, GEO	JH GEO I-5 (2010)	LK392505	LK392480
M. bernardi	Sra. de Gredos, E	AB 14996 (1992)	LN866960	LN866974
M. bernardi	Puerto de Cuarto Pelado, Valdelinares, E	JH 05 (2009)	LN866961	LN866975
M. stumperi	Briançon, F	AB 14128 (1989)	LN866962	
M. stumperi	Simplondorf, CH	AB 15754 (1999)	LN866963	
M. stumperi	Kastamonu, TR	AS 0498 (2012)	LN866964	
M. algerianus	Mts de Belezma, DZ	AB 12524 (1986)	LN866965	
M. gordiagini	Baška 1, Sv. Ivan, HR	J. Beibl K-37 (2005)	LK392507	LK392457
M. gordiagini	nr. Baška, HR	J. Beibl (2005)		LN866977
M. gordiagini	Nerezisca, HR	AS 0192 (2010)	LN866966	

[1] additional sequences for which currently no accession numbers are listed can be obtained from the authors
